# SARS-CoV-2 Infection and the Risk of New Chronic Conditions: Insights from a Longitudinal Population-Based Study

**DOI:** 10.3390/ijerph22020166

**Published:** 2025-01-26

**Authors:** David De Ridder, Anshu Uppal, Serguei Rouzinov, Julien Lamour, María-Eugenia Zaballa, Hélène Baysson, Stéphane Joost, Silvia Stringhini, Idris Guessous, Mayssam Nehme

**Affiliations:** 1 Geographic Information Research and Analysis in Population Health (GIRAPH) Lab, Department of Community Health and Medicine, Faculty of Medicine, 1205 Geneva, Switzerlandidris.guessous@hug.ch (I.G.); 2 Unit of Population Epidemiology, Division of Primary Care Medicine, Geneva University Hospitals, 1205 Geneva, Switzerlandmayssam.nehme@hug.ch (M.N.); 3 Geospatial Molecular Epidemiology (GEOME), Laboratory for Biological Geochemistry (LGB), School of Architecture, Civil and Environmental Engineering (ENAC), École Polytechnique Fédérale de Lausanne (EPFL), 1015 Lausanne, Switzerland; 4 La Source School of Nursing, University of Applied Sciences and Arts Western Switzerland (HES-SO), 1004 Lausanne, Switzerland; 5 School of Population and Public Health, and Edwin S.H, Leong Centre for Healthy Aging, Faculty of Medicine, University of British Columbia, Vancouver, BC V6T 1Z3, Canada; 6 Faculty of Medicine, University of Geneva, 1205 Geneva, Switzerland

**Keywords:** SARS-CoV-2, sociodemographic determinants, chronic conditions, mixed level modeling, geographically weighted regression, spatio-temporal analysis

## Abstract

Background: The post-acute impact of SARS-CoV-2 infections on chronic conditions remains poorly understood, particularly in general populations. Objectives: Our primary aim was to assess the association between SARS-CoV-2 infections and new diagnoses of chronic conditions. Our two secondary aims were to explore geographic variations in this association and to assess the association between SARS-CoV-2 infections and the exacerbation of pre-existing conditions. Methods: This longitudinal study used data from 8086 participants of the Specchio-COVID-19 cohort in the canton of Geneva, Switzerland (2021–2023). Mixed-effects logistic regressions and geographically weighted regressions adjusted for sociodemographic, socioeconomic, and healthcare access covariates were used to analyze self-reported SARS-CoV-2 infections, new diagnoses of chronic conditions, and the exacerbation of pre-existing ones. Results: Participants reporting a SARS-CoV-2 infection were more likely to be diagnosed with a new chronic condition compared to those who did not report an infection (adjusted odds ratio [aOR] = 2.15, 95% CI 1.43–3.23, adjusted *p*-value = 0.002). Notable geographic variations were identified in the association between SARS-CoV-2 infections and new diagnoses. While a positive association was initially observed between SARS-CoV-2 infections and exacerbation of pre-existing chronic conditions, this association did not remain significant after adjusting *p*-values for multiple comparisons. Conclusions: These findings contribute to understanding COVID-19’s post-acute impact on chronic conditions, highlighting the need for targeted health management approaches and calling for tailored public health strategies to address the pandemic’s long-term effects.

## 1. Introduction

The COVID-19 pandemic has profoundly impacted global health, extending far beyond acute illness and hospitalizations [1,2,3,4,5]. While extensive research has focused on the short-term effects of SARS-CoV-2 infections, including post-COVID conditions, the longer-term effects on chronic conditions remain to be fully understood [6,7,8]. Emerging evidence suggests that SARS-CoV-2 infections may be associated with a higher incidence of various chronic conditions, including diabetes [9], cardiovascular disease [10,11,12], and neurological disorders [13]. This potential double burden—consisting of the COVID-19 pandemic [5,14] and rising chronic conditions triggered by SARS-CoV-2 infections [15,16]—is particularly concerning given the pandemic’s scale.

The relationship between COVID-19 and chronic conditions is multifaceted. SARS-CoV-2 infection can lead to systemic inflammation and immune dysregulation, potentially leading to infection-related persistent symptoms and the development of other chronic conditions [9,10,11]. Concurrently, individuals with pre-existing chronic conditions face higher risks of severe COVID-19, leading to increased hospitalization and mortality rates [17,18]. This bidirectional relationship creates an interplay that intensifies the burden on healthcare systems [19].

Disruptions in the healthcare system, especially early in the pandemic (2020–2021), were linked to limited access to routine care services [20,21,22,23]. Social isolation [2], poor mental health [1,24,25], and decreased physical activity [26,27,28] due to pandemic-related restrictions have further exacerbated these issues. This complex interplay between the pandemic and individual health outcomes, particularly in relation to sociodemographic factors, has been demonstrated and extends to chronic conditions as well as health outcomes.

Indeed, social determinants of health, including income, education, and occupation, significantly influence both the risk of SARS-CoV-2 infection and the development and management of chronic conditions [19,29,30,31]. The pandemic has highlighted and often exacerbated existing health inequities [32,33], creating a syndemic—the interplay between co-present or sequential diseases and social and environmental factors that amplify negative health outcomes [24,31,34,35]. This phenomenon is especially concerning among already vulnerable populations and in disadvantaged areas [36,37].

While traditional epidemiological studies have identified these disparities, they most often rely on global models that assume uniform associations across geographic regions. However, the complex interplay between social determinants, SARS-CoV-2 infections, and chronic conditions may vary across space, necessitating approaches that can capture this spatial heterogeneity. Geospatial models have emerged as powerful tools to address this limitation, providing key insights into the spatial patterns and contextual factors underlying these disparities during the acute phase of the pandemic [38,39,40,41,42,43,44,45]. In the post-acute phase of the pandemic, these spatial methods can help elucidate whether vulnerable populations face an elevated risk of developing new chronic conditions, potentially further widening health inequities.

This study investigates the association between SARS-CoV-2 infections and chronic conditions in Geneva, Switzerland, through one primary objective and two secondary objectives. Our primary objective is to assess the association between SARS-CoV-2 infection and new chronic condition diagnoses. Secondarily, we aim to evaluate the presence of geographic variations (i.e., spatial heterogeneity) in this association, recognizing that the impact of SARS-CoV-2 infections may vary across different areas within the canton of Geneva. Additionally, we seek to examine the relationship between SARS-CoV-2 infections and the exacerbation of pre-existing chronic conditions. By addressing these objectives, we aim to provide insights into the health impacts of post-acute COVID-19, accounting for both the development of new chronic conditions and the exacerbation of existing ones, while considering the geographical nuances within our study area.

## 2. Methods

### 2.1. Study Design and Sample

This study is based on data from the Specchio-COVID19 digital cohort, a population-based digital study launched in December 2020 for the short- and long-term monitoring of the COVID-19 pandemic among the general population of the canton of Geneva, Switzerland. The details of the recruitment procedure have been described elsewhere [46]. Briefly, participants were recruited from seroprevalence surveys conducted on the general population between April 2020 and June 2022 [47,48,49,50] and a seroprevalence survey (SEROCoV-WORK+) conducted from May to September 2020, which targeted “essential” workers from public and private sectors likely mobilized during the initial lockdown [51]. From November 2020 onwards, participants who provided informed consent were invited to create an account on the Specchio-hub digital platform to complete a baseline questionnaire and receive periodic follow-up questionnaires. In 2021, 2022, and 2023 participants were invited to complete annual general health follow-up questionnaires. These questionnaires covered various aspects of health including general and mental health, chronic diseases, COVID-19 infections, symptoms, and other relevant topics. This approach provided longitudinal data on health outcomes. All data were collected through the secure Specchio-hub platform, ensuring the proper management and storage of sensitive data.

The study was approved by the Cantonal Research Ethics Commission of Geneva, Switzerland (nº 2020-00881).

### 2.2. Inclusion Criteria

Preliminary inclusion criteria in this study required participants to reside within the canton of Geneva and report their sex as either “Male” or “Female”. We excluded those who reported their sex as “Other” due to insufficient case numbers for meaningful analysis. To ensure privacy preservation in spatial analyses, participants needed to have at least one neighbor within a radius of 800 m; geographically isolated individuals were excluded. Among those meeting these preliminary criteria, final inclusion in the analysis required completion of the baseline questionnaire and at least one of the three follow-up questionnaires administered in 2021, 2022, or 2023.

### 2.3. Measures

#### 2.3.1. Chronic Conditions and SARS-CoV-2 Infections

At inclusion, participants self-reported pre-existing chronic conditions: “Do you suffer from a chronic illness that may require regular care or treatment?”. The complete list and definitions of chronic conditions considered in this study is available in Table S1.

At each annual follow-up, participants reported significant health events experienced over the past period using the following question: “Over the past twelve (six in 2021) months, have you experienced one or more significant health events?”. Participants reported on the specific type of health events from a predefined list. For SARS-CoV-2 infections, participants were specifically asked to only report confirmed infections: “Confirmed diagnosis of COVID-19 disease through positive PCR test, positive rapid antigen test, or positive self-test, with or without symptoms ?”). New chronic condition diagnoses were captured through the question “Newly diagnosed disease or allergy, excluding COVID-19 disease ?”, and exacerbations of pre-existing conditions through “Worsening of a known chronic disease ?”.

This approach allowed us to track SARS-CoV-2 infections and both the diagnosis of new chronic conditions and the exacerbation of existing ones throughout the study period (Table S2).

To account for the variation in the reporting period between 2021 and subsequent years, we included the study year as a variable in our statistical models. Additionally, we introduced a variable that captures the time (in years) between two consecutive follow-ups. This adjustment helps manage potential overlaps where the 12-month periods from successive follow-ups might not align perfectly. For instance, an individual participating at the end of one year and at the beginning of the next could have overlapping months in their reported data. By incorporating these time-related variables, we aimed to control for these temporal inconsistencies and ensure a more accurate analysis of the longitudinal data.

#### 2.3.2. Covariates

Sociodemographic factors included age group (18–34, 35–49, 50–64, 65–79, 80+), biological sex (male, female) and nationality (Swiss, other). Socioeconomic factors included education level, employment status, household income and living status (alone, single parent with or without children, with other adults). Healthcare access factors included health insurance deductible (300, 500, 1000, 1500, 2000, 2500 CHF, Don’t know/Don’t wish to answer) and forgoing healthcare within the last 12 months (Yes/No).

Education level was categorized as primary (none or compulsory education), secondary (high school diploma or vocational training), or tertiary (university level qualification). Employment status was categorized as employed or self-employed, retired, unemployed, or other economically inactive (not working and not looking for work, such as students, and people unable to work for health reasons or disability). Household income was assessed by asking participants the range of income that best represents their family’s gross income (before deducting social security contributions and taxes) in the last year. That is, the sum of the income of everyone in the household, or the participant’s own income if living alone. We then used the reported income range and the living status to define categories of household income: low, middle, high, or “don’t know/don’t wish to answer”.

#### 2.3.3. Residential Address

Participant residential addresses were geocoded using the Swiss Federal Administration geographical information platform [52], which enables matching addresses against a comprehensive national dataset. We excluded 61 participants whose address could not be geocoded and five geographically isolated participants (no neighbor within 800 m) for privacy preservation.

### 2.4. Statistical Modeling

We used generalized linear mixed-effects modeling (GLMM) to investigate the relationship between SARS-CoV-2 infections and health events (new chronic condition diagnoses or exacerbation of pre-existing conditions) [53]. GLMM was chosen for its ability to handle binary outcomes and the hierarchical structure of our data. Models were fitted using the glmer function (lme4 package version 1.1.35.4) in R (version 4.3.2) [54], specifying a binomial family with a logit link function.

We addressed missing item responses in covariates using Multiple Imputation by Chained Equations (mice) [55]. The imputation process included primary exposures, covariates and outcomes. We used predictive mean-matching for normally distributed variables, logistic regression for binary variables, and polytomous regression for categorical variables. Five imputed datasets were generated for analysis robustness.

We employed several strategies to account for potential bias from differential participation patterns. First, we categorized participation patterns into three groups: complete follow-up (all three follow-ups completed), dropout (missed the final follow-up), and intermittent missing (present in the final follow-up but missed intermediate ones). We then incorporated these patterns as a covariate and tested their influence through an interaction term with SARS-CoV-2 infection, as detailed in Model 2 below.

To account for repeated measurements and temporal dependencies, we incorporated random effects for the unique identifier of participants. We used the BOBYQA (Bound Optimization BY Quadratic Approximation) optimization algorithm [53,56] for model fitting. We assessed multicollinearity using variance inflation factors (VIF) on each imputed dataset, with no values exceeding the problematic threshold of 10.

We implemented a sequential modeling strategy to examine the associations between primary exposure and each outcome (i.e., new chronic condition diagnoses or exacerbation of pre-existing conditions), progressively adjusting for different sets of covariates (as detailed in Section 2.3.2. Covariates). We considered 5 models of increasing complexity: model 1 (base) including primary exposure, time (follow-up year) and random effect; model 2, additionally adjusted for “methodological” variables including an interaction between the primary exposure and the participation pattern (complete, dropout, intermittent missing), time in years between two consecutive follow-ups and an interaction between the primary exposure and the presence of a chronic disease at baseline (Yes/No); model 3, additionally adjusted for sociodemographic covariates; model 4, additionally adjusted for socioeconomic covariates; and model 5, additionally adjusted for healthcare access covariates.

This sequential approach allowed us to assess the stability of the main effect across different levels of adjustment and evaluate the impact of each set of factors on the primary association.

### 2.5. Pooling of Results

We pooled results from the imputed datasets using Rubin’s rules as implemented in the mice package [55]. This pooling process combines coefficients and standard errors from each imputed dataset, accounting for within- and between-imputation variability. The pooled estimates provide a comprehensive summary of effect sizes and their uncertainties, appropriately handling the uncertainty introduced by the imputation process. We report the pooled adjusted odds ratios (aORs) for our primary exposure, the interaction between participation pattern and our primary exposure and forgoing healthcare. All reported *p*-values were adjusted for multiple comparisons using the Benjamini–Hochberg method to control the false discovery rate (FDR) [57].

### 2.6. Spatial Heterogeneity

To investigate spatial heterogeneity in the association between SARS-CoV-2 infections and new chronic condition diagnoses, we used binomial logistic Geographically Weighted Regressions (GWR) for each follow-up [58]. We used the fully adjusted modeling specification including all covariates used in the previous mixed-effects models and employed a bisquare kernel with a k-nearest neighbors (KNN) bandwidth of 512 neighbors. The bisquare kernel was chosen for its smooth, continuous weighting that gradually diminishes to zero, reducing the influence of distant observations [59]. We opted for a manual specification of the bandwidth rather than relying on a model fit criterion because our objective was to evaluate the existence of spatial heterogeneity [59]. The choice of 512 nearest neighbors ensured a sufficiently large bandwidth to detect meaningful spatial patterns while avoiding over-smoothing. This approach allowed for a more controlled assessment of this association’s spatial heterogeneity, prioritizing the detection of localized variations over overall model fit optimization. GWR analyses were performed in Python (version 3.11) using the mgwr package [59] (version 2.2.1).

## 3. Results

### 3.1. Descriptive Analyses

Of the 12,459 individuals who participated in the inclusion questionnaire, 11,700 (93.9%) met the preliminary inclusion criteria as defined in the Methods section. Among these eligible participants, 8086 (69.1%) completed at least one follow-up questionnaire, forming the basis for our analyses. Follow-up participation rates were 52.4%, 51.1%, and 42.3% for the first, second, and third follow-ups, respectively, totaling 17,053 observations.

Participation patterns among the 8086 participants were: 42.9% (*n* = 3467) completed all follow-ups, 18.3% (*n* = 1481) had intermittent missing data, and 38.8% (*n* = 3138) dropped out (missed the final follow-up, including those absent for both second and final).

Most time-invariant sample demographics remained stable throughout the study period, except for age, work situation, and living status, with the proportion of older, retired individuals logically increasing over time (Table 1). The most represented age category was 50–64 (37%, median age: 53 years), and there were more women (59%) than men. Most had tertiary education (65%) and were salaried workers (63%). The sample was predominantly Swiss nationals (83%), with many living with a partner, either with (43%) or without (28%) children. For time-invariant health-related factors, 26% reported having a chronic condition and 8.5% reported forgoing healthcare at baseline.

Time-varying variables showed significant differences across years. New diagnoses increased from 1.3% (*n* = 82) in 2021 to 2.4% (*n* = 119) in 2023 (*p* < 0.001). Exacerbated pre-existing chronic conditions were reported by 0.9% (*n* = 53) in 2021, 1.3% (*n* = 76) in 2022, and 1.2% (*n* = 61) in 2023. Self-reported SARS-CoV-2 infections peaked in 2022 (19%, *n* = 1131), then decreased to 12% (*n* = 589) in 2023. When stratified by SARS-CoV-2 infection status, new chronic condition diagnoses were more frequent among infected participants, particularly in later years (2021: 2.3% vs. 1.3%, *p* = 0.2; 2022: 3.3% vs. 1.5%, *p* < 0.001; 2023: 4.8% vs. 2.1%, *p* < 0.001). Similarly, exacerbation of pre-existing conditions was generally more common among those with SARS-CoV-2 infections (2021: 1.9% vs. 0.8%, *p* = 0.075; 2022: 1.9% vs. 1.1%, *p* = 0.025; 2023: 2.0% vs. 1.1%, *p* = 0.059) (Table S2).

Baseline characteristics differed significantly across participation patterns (complete, dropout, intermittent missing) for most variables, except sex and forgoing healthcare (Table S3). These differences were considered in subsequent modeling analyses by including participation patterns as a covariate in all model specifications except the simplest “Base” model, testing for effect modification through an interaction term between participation patterns and SARS-CoV-2 infection and adjusting for all baseline characteristics that differed across patterns.

### 3.2. Determinants of New Chronic Condition Diagnosis

**Figure 1:**
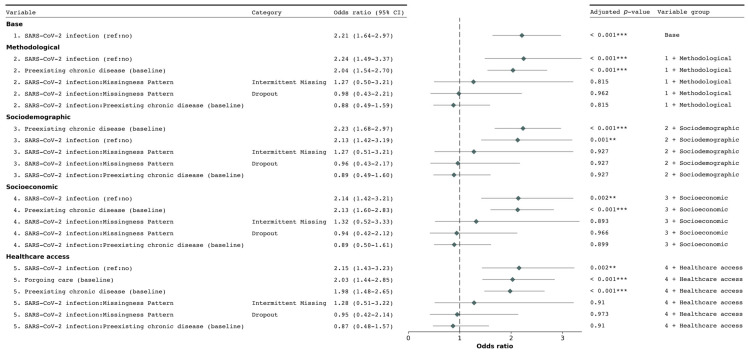
Forest plot of odds ratios for the association between SARS–CoV–2 infections and new chronic condition diagnoses. The plot presents adjusted odds ratios and 95% confidence intervals from the generalized linear mixed-effects models (Model 1 to 5). Odds ratios greater than 1 indicate increased odds of the outcome, while those less than 1 indicate decreased odds. *p*–values were adjusted using the False Discovery Rate (FDR) method to account for multiple comparisons. The dotted vertical line represents an odds ratio of 1 (no association). Statistical significance: ** *p* < 0.01, *** *p* < 0.001.

Figure 1 presents the results of the mixed-effects logistic regression sequential models, evaluating the association between SARS-CoV-2 infections and new chronic condition diagnoses.

In all analyses, participants who reported a SARS-CoV-2 infection had substantially higher odds of being diagnosed with a new chronic condition than those who did report an infection. The strength of this association varied across models, with the aOR ranging from 2.21 (95% CI 1.64–2.97, adjusted *p*-value < 0.001) in the base model (model 1) to 2.15 (95% CI 1.43–3.23, adjusted *p*-value < 0.001) in the fully adjusted model (model 5). Notably, the association slightly increased from model 1 to model 2 after adding “methodological” covariates, but subsequently weakened as additional covariates were introduced in models 3 through 5.

Examining participation patterns, participants who dropped out or had intermittent missing data were significantly less likely to report new chronic condition diagnoses across all models. In the fully adjusted model, the aOR for dropout was 0.533 (95% CI 0.36–0.79, adjusted *p*-value = 0.013), while for intermittent missing data, it was 0.358 (95% CI 0.22–0.59, adjusted *p*-value < 0.001). However, the interaction between participation patterns and the primary exposure was not statistically significant in any model (e.g., in model 5: aOR 0.95, 95% CI 0.42–2.14, adjusted *p*-value = 0.973). This suggests that while participation patterns affect the likelihood of new diagnoses, they do not significantly modify the effect of SARS-CoV-2 infection on new chronic condition diagnoses.

Several other factors showed significant associations with new chronic condition diagnoses. Individuals who reported forgoing healthcare in the inclusion questionnaire had higher odds of new diagnoses (aOR 2.03, 95% CI 1.44–2.85, adjusted *p*-value < 0.001 in model 5). The presence of a chronic condition at baseline was strongly associated with increased odds of a new chronic condition diagnosis in all models (e.g., aOR 1.98, 95% CI 1.48–2.65, adjusted *p*-value < 0.001 in model 5), highlighting the importance of considering pre-existing health conditions in assessing the risk of developing new chronic conditions.

To evaluate whether individuals already burdened by chronic conditions may be at increased risk of developing additional conditions due to SARS-CoV-2 infections, we included an interaction effect between SARS-CoV-2 infections and preexisting chronic conditions. This interaction showed a non-significant association with new diagnoses in all models (e.g., aOR 0.87, 95% CI 0.48–1.57, adjusted *p*-value = 0.91 in model 5).

**Figure 2:**
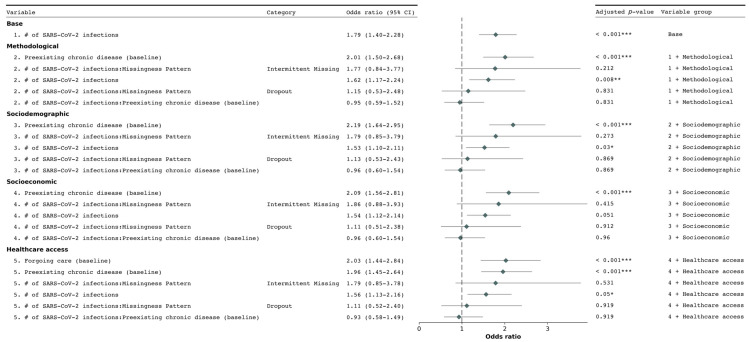
Forest plot of odds ratios for the association between the cumulated number of SARS–CoV–2 infections and new chronic condition diagnoses. The plot presents adjusted odds ratios and 95% confidence intervals for the generalized linear mixed–effects models (Model 1 to 5). Odds ratios greater than 1 indicate increased odds of the outcome, while those less than 1 indicate decreased odds. *p*–values were adjusted using the False Discovery Rate (FDR) method to account for multiple comparisons. The dotted vertical line represents an odds ratio of 1 (no association). Statistical significance: * *p* < 0.05, ** *p* < 0.01, *** *p* < 0.001.

To investigate the potential cumulative effect of repeated SARS-CoV-2 infections, we assessed two additional model specifications. In the first specification, we considered the cumulative number of infections at each follow-up as a primary exposure. We observed significantly increased odds of new chronic condition diagnoses associated with the cumulated number of SARS-CoV-2 infections in models 1, 2, 3, and 5 (Figure 2). In model 4, which additionally included socioeconomic factors, the association did not remain significant after adjusting the *p*-value for multiple comparisons (adjusted *p*-value > 0.05).

The second specification incorporated both the binary variable indicating SARS-CoV-2 infection status and the cumulative number of reported infections at each follow-up. In this specification, while the binary infection status remained significantly associated with new chronic condition diagnoses in models 1 to 4, in model 5 it showed an aOR of 1.92 (95% CI 1.48–2.50) with an adjusted *p*-value of 0.055. The cumulative number of infections showed no significant independent association across all models (Figure S1). Our variance inflation factor (VIF) evaluation indicated no significant collinearity issues.

Additionally, we examined whether SARS-CoV-2 infections were associated with a worsening of pre-existing conditions among 1960 participants who reported having a chronic condition either at baseline or in the previous follow-up (4168 total observations). While SARS-CoV-2 infection showed positive associations with condition exacerbation across all models (*p* < 0.05), after FDR correction, only the base model retained statistical significance (aOR 2.11, 95% CI 1.24–3.59, adjusted *p*-value = 0.012). Detailed results are presented in Figure S2.

### 3.3. Spatial Heterogeneity in the Association Between New Diagnosis of Chronic Conditions and SARS-CoV-2 Infections

**Figure 3:**
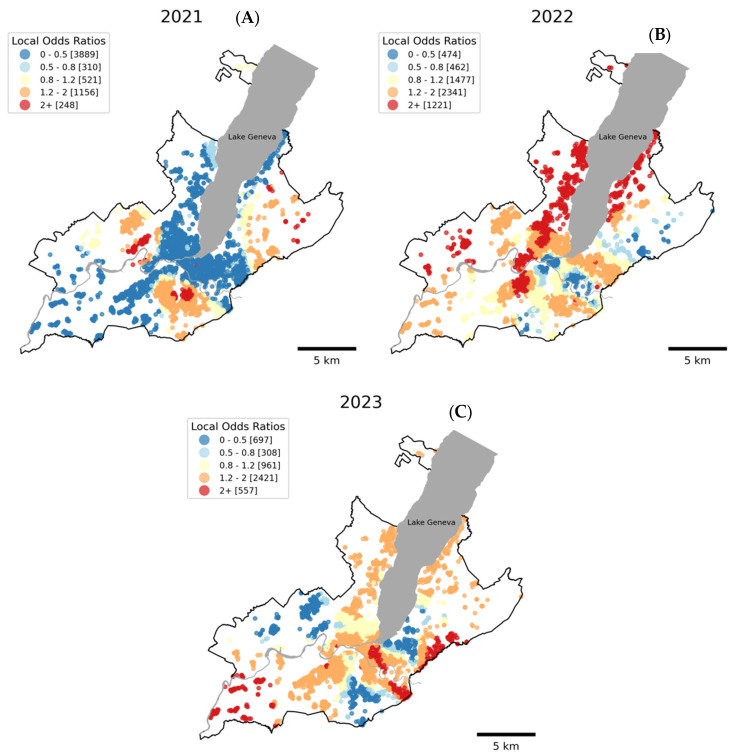
Local odds ratio for the association between SARS-CoV-2 infections and new diagnoses of a chronic condition using geographically weighted regression (GWR) across three years: (**A**) 2021, (**B**) 2022 and, (**C**) 2023. Each dot on the map represents a participant’s location. The color scale indicates local odds ratios: dark blue (0–0.5), light blue (0.5–0.8), yellow (0.8–1.2), orange (1.2–2), and red (2+). Numbers in brackets show the count of participants in each category. Areas with odds ratios greater than 1 (orange and red) suggest a higher likelihood of new chronic condition diagnoses associated with a SARS-CoV-2 infection, while areas with odds ratios less than 1 (blue shades) suggest a lower likelihood.

The GWR analyses conducted for each of the three follow-ups (2021, 2022, and 2023) returned local odds ratios (LORs) for the relationship between SARS-CoV-2 infections and the new diagnoses of chronic conditions (Figure 3). There was marked spatial heterogeneity in LORs across all three years. In 2021, a distinct pattern emerged with lower LORs (depicted in blue) predominating in the central and western portions of the canton of Geneva (Figure 3A). A notable shift in the spatial pattern is evident as time progresses from the first to the third follow-up (Figure 3A–C). Some areas, particularly in the east and center, showed consistently high odds ratios across all follow-ups while other areas had changing patterns that initially showed lower odds (blue) in the first follow-up and later showed higher odds (red) in subsequent follow-ups.

## 4. Discussion

### 4.1. Main Findings

This study shows that SARS-CoV-2 infections were associated with higher odds of being diagnosed with a new chronic condition, with a dose–response relationship when considering cumulative SARS-CoV-2 infections. Notably, this association only slightly weakened after sequential adjustment for sociodemographic, socioeconomic, and health access factors. While both SARS-CoV-2 infection and pre-existing chronic conditions independently increased the risk of diagnoses, we found no significant interaction between these two specific factors. Additionally, individuals who reported forgoing healthcare showed higher odds of new diagnoses. Our geographically weighted regression analysis revealed that some populations within Geneva may be at higher risk of post-acute sequelae, underlining the importance of considering local contexts in health outcomes.

### 4.2. Comparison with Existing Literature

Our findings align with and extend previous research on post-acute COVID-19 sequelae. While most studies have focused on severe outcomes during the acute phase or examined specific populations, our study provides insights into the general population over an extended period.

The increased odds of developing a new chronic condition diagnosis are in line with the findings from studies on veterans [10,11], hospitalized individuals [60], and specific health insurance cohorts [12,13]. The results of our analyses provide new information on a population-based level, and in mostly outpatient individuals. These new results suggest the importance of looking further into the direct and indirect effects of COVID-19 in the development of chronic conditions.

The association between SARS-CoV-2 infection and new chronic conditions showed modest attenuation after adjustment for sociodemographic, socioeconomic, and healthcare access factors (from aOR 2.24 in model 2 to aOR 2.15 in the fully adjusted model). This pattern suggests that these factors may influence the relationship between SARS-CoV-2 infection and new chronic conditions, though they do not fully account for the observed association. Social determinants of health and healthcare access patterns could play a role in both SARS-CoV-2 infection detection and chronic condition diagnosis. However, the persistence of a strong association after adjustment, even in our most complex model specification, suggests that SARS-CoV-2 infection maintains an independent relationship with new chronic conditions. Future research might explore additional model specifications, including potential non-linear relationships and interactions between these factors.

The unadjusted *p*-values suggested a potential association between SARS-CoV-2 infections and exacerbation of pre-existing chronic conditions. However, this relationship did not remain statistically significant after adjusting for multiple comparisons, potentially due to power issues in the subsample used in this analysis.

This finding, while aligning with the known impacts of viral infections on chronic conditions [61,62,63], should be interpreted cautiously and merits further investigation in larger samples. The potential exacerbation could be related to the SARS-CoV-2 infection itself but also to the disturbances in access to care. Indeed, we found a strong association between forgoing healthcare and adverse health outcomes, highlighting the indirect effects of the pandemic through disruptions in the healthcare system, consistent with previous findings [20,21,22,23].

The analysis of the interplay between SARS-CoV-2 infection and pre-existing chronic conditions yielded nuanced insights. While both factors were independently associated with an increased risk of developing new chronic conditions, we found no significant interaction between them. This suggests that the presence of pre-existing conditions does not substantially amplify the risk of new chronic condition diagnoses following SARS-CoV-2 infection beyond the additive effects of each factor. Furthermore, our examination of cumulative SARS-CoV-2 infections revealed a potential dose–response relationship when modeled alone. However, this effect was not significant when considered alongside binary infection status, suggesting that the occurrence of infection, rather than the number of repeat infections, may be more predictive of new chronic condition diagnoses. These findings underline the complexity of COVID-19’s post-acute health impacts and highlight the need for careful consideration of how SARS-CoV-2 exposure is operationalized in future studies.

The GWR analyses complement our GLMM findings by revealing the spatial heterogeneity in the association between SARS-CoV-2 infections and new chronic condition diagnoses. While the GLMM results demonstrate an overall positive association, the GWR analyses show that this relationship varies meaningfully across Geneva’s geography, with most areas showing odds ratios above 1 in 2022 and 2023. In some areas, however, local odds ratios (LORs) were below 1, indicating high SARS-CoV-2 infection rates coincided with low rates of new chronic condition diagnoses. These contrasting local patterns, together with the predominant positive association, demonstrate substantial geographic variation in post-acute COVID-19 impacts, extending previous research on COVID-19 outcome inequalities [17,32,36,39,64].

These patterns likely reflect a complex interplay of factors beyond those included in our models. These may include population density and residential overcrowding, differential access to healthcare services [20,21,22,23], variations in vaccination uptake [65,66], or environmental factors such as air pollution. Indeed, air pollution, known to vary geographically across Geneva [64] exacerbates both COVID-19 severity and chronic condition development [64,67,68,69]. Moreover, the increase in air pollution back towards pre-pandemic levels from 2021 to 2023, as seen in other regions, may be playing a potential role in the temporal trend observed in the GWR analyses. Additionally, while population density and residential overcrowding primarily affect viral transmission rates [70], they may also indirectly influence the association between SARS-CoV-2 infection and chronic condition development through factors such as increased viral load [71] and prolonged exposure [64].

The temporal trend observed in the GWR analyses, showing an increasing proportion of LORs above 1 from 2021 to 2023, suggests potential temporal lag effects in the development or diagnosis of chronic conditions following SARS-CoV-2 infection and may reflect the progressive impact of vaccination rollout [65,66]. Areas with negative associations (LORs < 1), notably in 2021, may result from these lag effects or unmeasured differences in healthcare utilization that persist after adjustment for socioeconomic, sociodemographic, and healthcare access factors while areas showing consistently high odds ratios across follow-ups are of particular concern and warrant further investigation. These geographical and temporal considerations underscore the importance of long-term follow-up studies to fully understand the chronic health impacts of COVID-19 and tailored, localized public health interventions and resource allocation in the post-pandemic era.

### 4.3. Strengths and Limitations

Our study has several notable strengths. The longitudinal design, spanning three years, allowed us to observe temporal trends in the relationship between SARS-CoV-2 infections and chronic conditions. This extended timeframe provides insights into the health impacts of COVID-19 over a three-year period, a critical aspect often overlooked in shorter-term studies. Our comprehensive questionnaires captured a wide array of sociodemographic and healthcare access determinants, enabling a nuanced analysis of factors influencing chronic condition development and exacerbation. The geocoding of participants’ addresses facilitated spatial analysis, revealing important geographic variations in COVID-19’s post-acute impact. Additionally, our study included both new diagnoses and the exacerbation of existing conditions, providing a more complete picture of COVID-19’s chronic health consequences.

However, several limitations should be considered when interpreting our results. The reliance on self-reported data may introduce recall bias, particularly for SARS-CoV-2 infections and chronic condition diagnoses. While we specifically asked for confirmed SARS-CoV-2 infections, the accuracy of self-reported health information remains a concern. It is worth noting that while our exposure measurement is imperfect, alternative methods such as seroprevalence studies would not provide a complete solution as they often lack information on infection timing and re-infections [72]. On the other hand, SARS-CoV-2 testing data from testing centers may suffer from even greater underreporting [73].

Our study design, with annual follow-ups, limited our ability to establish precise temporal sequences between SARS-CoV-2 infections and chronic condition diagnoses within each 12-month period. Although the multi-year design allows us to observe broader temporal patterns and trends, this temporal ambiguity calls for caution in interpreting causal relationships. Furthermore, the increased diagnosis of chronic conditions among individuals with SARS-CoV-2 infections may partly reflect increased medical attention and screening related to their COVID-19 experience, potentially leading to healthcare utilization bias. Additionally, our analysis considered all chronic conditions together, which limits our ability to disentangle associations by specific disease categories. This aggregation may obscure the potentially varied effects of SARS-CoV-2 on different types of chronic conditions. Lastly, the reduction in sample size over the three-year period may introduce sampling bias, as participants with certain health conditions might be more or less likely to continue participating. While we employed statistical techniques to mitigate this, including pattern-mixture modeling, the potential for bias remains a limitation of our study.

## 5. Conclusions

This three-year longitudinal study provides insights into the association between SARS-CoV-2 infections and the onset of new chronic conditions, with evidence of a dose–response relationship. We identified substantial geographic variations within the canton of Geneva, highlighting the need for localized post-pandemic health management approaches. These findings contribute to a better understanding of COVID-19’s post-acute impact and call for tailored public health strategies and continued long-term studies to address the lasting impacts of COVID-19 on chronic conditions.

## Figures and Tables

**Table 1:** Baseline sociodemographic characteristics and time-variant health events at follow-ups.

Variable	Overall, *n*= 17,053 ^1^	2021, *n* = 6127 ^1^	2022, *n* = 5978 ^1^	2023, *n* = 4948 ^1^	*p*-Value ^2^
Age—Baseline					<0.001
18–34	2073 (12%)	869 (14%)	666 (11%)	538 (11%)	
35–49	5626 (33%)	2058 (34%)	2000 (33%)	1568 (32%)	
50–64	6227 (37%)	2197 (36%)	2176 (36%)	1854 (37%)	
65–79	2909 (17%)	944 (15%)	1036 (17%)	929 (19%)	
80+	218 (1.3%)	59 (1.0%)	100 (1.7%)	59 (1.2%)	
Sex—Baseline					0.2
Female	10,028 (59%)	3547 (58%)	3543 (59%)	2938 (59%)	
Male	7025 (41%)	2580 (42%)	2435 (41%)	2010 (41%)	
Insurance deductible—Baseline					>0.9
Missing ^3^	4 (<0.1%)	1 (<0.1%)	1 (<0.1%)	2 (<0.1%)	
300 CHF	5829 (34%)	2058 (34%)	2070 (35%)	1701 (34%)	
500 CHF	2908 (17%)	1046 (17%)	1024 (17%)	838 (17%)	
1000 CHF	693 (4.1%)	252 (4.1%)	237 (4.0%)	204 (4.1%)	
1500 CHF	1277 (7.5%)	461 (7.5%)	442 (7.4%)	374 (7.6%)	
2000 CHF	364 (2.1%)	136 (2.2%)	126 (2.1%)	102 (2.1%)	
2500 CHF	4772 (28%)	1730 (28%)	1648 (28%)	1394 (28%)	
Don’t know/don’t wish to answer	873 (5.1%)	326 (5.3%)	310 (5.2%)	237 (4.8%)	
No Swiss health insurance	333 (2.0%)	117 (1.9%)	120 (2.0%)	96 (1.9%)	
Education level—Baseline					0.3
Missing ^3^	2 (<0.1%)	0 (0%)	1 (<0.1%)	1 (<0.1%)	
Other	24 (0.1%)	5 (<0.1%)	13 (0.2%)	6 (0.1%)	
Primary	666 (3.9%)	240 (3.9%)	252 (4.2%)	174 (3.5%)	
Secondary	5366 (31%)	1941 (32%)	1894 (32%)	1531 (31%)	
Tertiary	10,995 (64%)	3941 (64%)	3818 (64%)	3236 (65%)	
Work situation—Baseline					<0.001
Missing ^3^	4 (<0.1%)	1 (<0.1%)	1 (<0.1%)	2 (<0.1%)	
Freelance/sole trader	1247 (7.3%)	449 (7.3%)	435 (7.3%)	363 (7.3%)	
Other economically inactive	1416 (8.3%)	530 (8.7%)	485 (8.1%)	401 (8.1%)	
Retired	3294 (19%)	1046 (17%)	1199 (20%)	1049 (21%)	
Salaried	10,659 (63%)	3942 (64%)	3712 (62%)	3005 (61%)	
Unemployed	433 (2.5%)	159 (2.6%)	146 (2.4%)	128 (2.6%)	
Household income—Baseline					0.4
Missing ^3^	10 (<0.1%)	1 (<0.1%)	6 (0.1%)	3 (<0.1%)	
Don’t know/don’t wish to answer	2988 (18%)	1096 (18%)	1050 (18%)	842 (17%)	
High	2520 (15%)	888 (14%)	891 (15%)	741 (15%)	
Low	2348 (14%)	860 (14%)	846 (14%)	642 (13%)	
Middle	9187 (54%)	3282 (54%)	3185 (53%)	2720 (55%)	
Occupation—Baseline					0.059
Missing ^3^	12 (<0.1%)	3 (<0.1%)	4 (<0.1%)	5 (0.1%)	
Blue collar workers	1481 (8.7%)	564 (9.2%)	540 (9.0%)	377 (7.6%)	
Higher-grade white-collar workers	4442 (26%)	1596 (26%)	1530 (26%)	1316 (27%)	
Independent workers	310 (1.8%)	90 (1.5%)	121 (2.0%)	99 (2.0%)	
Lower-grade white collar workers	4373 (26%)	1557 (25%)	1549 (26%)	1267 (26%)	
Other	869 (5.1%)	319 (5.2%)	303 (5.1%)	247 (5.0%)	
Professional-Managers	5566 (33%)	1998 (33%)	1931 (32%)	1637 (33%)	
Nationality—Baseline					0.11
Missing ^3^	1 (<0.1%)	0 (0%)	1 (<0.1%)	0 (0%)	
Foreigner	2846 (17%)	1037 (17%)	1029 (17%)	780 (16%)	
Swiss	14,206 (83%)	5090 (83%)	4948 (83%)	4168 (84%)	
Living status—Baseline					0.015
Missing ^3^	5 (<0.1%)	0 (0%)	3 (<0.1%)	2 (<0.1%)	
Cohabitation	1152 (6.8%)	470 (7.7%)	374 (6.3%)	308 (6.2%)	
Single	2603 (15%)	925 (15%)	918 (15%)	760 (15%)	
Single parent	1150 (6.7%)	417 (6.8%)	392 (6.6%)	341 (6.9%)	
With partner and kids	7368 (43%)	2661 (43%)	2616 (44%)	2091 (42%)	
With partner, without kids	4775 (28%)	1654 (27%)	1675 (28%)	1446 (29%)	
Chronic condition—Baseline	4426 (26%)	1537 (25%)	1571 (26%)	1318 (27%)	0.14
Missing ^3^	2 (<0.1%)	1 (<0.1%)	1 (<0.1%)	0 (0%)	
Forgoing healthcare—Baseline	1447 (8.5%)	512 (8.4%)	515 (8.6%)	420 (8.5%)	0.9
Missing ^3^	3 (<0.1%)	0 (0%)	1 (<0.1%)	2 (<0.1%)	
Health event—New diagnosis	313 (1.8%)	82 (1.3%)	112 (1.9%)	119 (2.4%)	<0.001
Health event—Worsening conditions	190 (1.1%)	53 (0.9%)	76 (1.3%)	61 (1.2%)	0.066
Health event—SARS-CoV-2 infection	1982 (12%)	262 (4.3%)	1131 (19%)	589 (12%)	<0.001

^1^ n (%), ^2^ Pearson’s Chi-squared test, ^3^ Missing items were imputed using multiple imputation with chained equations (mice).

## Data Availability

Study data that underlie the results reported in this article can be made available to the scientific community after deidentification of individual participants, and upon submission of a data request application to the investigator board via the corresponding author.

## Supplementary Materials

The following supporting information can be downloaded at: https://www.mdpi.com/article/10.3390/ijerph22020166/s1, Figure S1: Forest plot of odds ratios for factors associated with new chronic condition diagnosis; Figure S2. Forest plot of odds ratios for the association between SARS-CoV-2 infections and worsening chronic conditions; Table S1: List of chronic conditions presented to participants at inclusion and follow-ups; Table S2: Distribution of new chronic condition diagnoses and worsening of pre-existing conditions by SARS-CoV-2 infection status and year of follow-up; Table S3: Comparison of baseline characteristics across participation patterns.
