# Supplementary material for: SARS-CoV-2 Infection and the Risk of New Chronic Conditions: Insights from a Longitudinal Population-Based Study
Source: Int J Environ Res Public Health. 2025 Jan 26;22(2):166. doi: 10.3390/ijerph22020166 (PMC11855532; doi:10.3390/ijerph22020166)
Supplement: Supplementary file 1 [file ijerph-22-00166-s001.zip › ijerph-3356204-supplementary.pdf]

Supplementary material

Original (French)	Translated (English)	Category	Subcategory	Variable type
**Souffrez-vous d'une maladie chronique qui peut nécessiter des soins ou un traitement régulier ?**	Do you suffer from a chronic illness that may require regular care or treatment?			factor
**Hypertension artérielle**	Hypertension	Cardiovascular	High blood pressure	logical
**Diabète**	Diabetes	Metabolic	Diabetes	logical
**Dyslipidémie: hypercholestérolémie « cholestérol », hypertriglycéridémie**	Dyslipidemia: hypercholesterolemia "cholesterol", hypertriglyceridemia	Metabolic	Dyslipidemia	logical
**Obésité**	Obesity	Metabolic	Obesity	logical
**Maladie cardio-vasculaire (angine de poitrine, antécédent d'infarctus, AVC, ...)**	Cardiovascular disease (angina pectoris, previous heart attack, stroke, etc.)	Cardiovascular	Cardiovascular (except HBP)	logical
**Maladies chroniques des voies respiratoires (bronchite chronique, BPCO, asthme...)**	Chronic respiratory diseases (chronic bronchitis, COPD, asthma, etc.)	Respiratory		logical
**Allergies**	Allergies	Allergies		logical
**Problèmes articulaires, rhumatismes**	Joint problems, rheumatism	Joint		logical
**Maladies musculaires ou squelettiques**	Muscular or skeletal diseases	Musculoskeletal/Myopathy	Musculoskeletal/Myopathy	logical
**Ostéoporose**	Osteoporosis	Musculoskeletal/Myopathy	Osteoporosis	logical
**Troubles digestifs**	Digestive disorders	Gastrointestinal		logical
**Troubles gynécologiques**	Gynecological disorders	Gynecological		logical
**Migraines**	Migraines	Neurological		logical
**Maladies neurologiques**	Neurological diseases	Neurological		logical
**Maladies chroniques du foie**	Chronic liver disease	Gastrointestinal		logical
**Maladies rénales chroniques**	Chronic kidney disease	Renal		logical

**Problème de thyroïde: hypo/hyperthyroïdie**	Thyroid problems: hypo/hyperthyroidism	Metabolic	logical
**VIH**	HIV	Infectious	logical
**Autres maladies infectieuses (hépatite B, hépatite C, tuberculose)**	Other infectious diseases (hepatitis B, hepatitis C, tuberculosis)	Infectious	logical
**Cancer actif ou sous traitement (chimiothérapie, radiothérapie ou immunothérapie).**	Active cancer or cancer under treatment (chemotherapy, radiotherapy or immunotherapy).	Cancer	logical
**Greffe d'organe, greffe de moelle osseuse**	Organ transplants, bone marrow transplants	Other	logical
**Troubles de l'immunité dus à un traitement ou à une maladie**	Immunity disorders due to treatment or disease	Immune	logical
**Trouble de l'humeur, y compris trouble dépressif majeur et trouble bipolaire**	Mood disorders, including major depressive disorder and bipolar disorder	Mood	logical
**Trouble d'anxiété, y compris le trouble d'anxiété généralisée et le trouble d'anxiété sociale**	Anxiety disorders, including generalized anxiety disorder and social anxiety disorder	Anxiety	logical
**Schizophrénie ou autre trouble psychotique**	Schizophrenia or other psychotic disorders	Psychosis	logical
**Addictions (alcool, drogues, jeux...)**	Addictions (alcohol, drugs, gambling, etc.)	Addiction	logical
**Maladies dermatologiques (maladies de la peau)**	Dermatological diseases (skin disorders)	Dermatological	logical
**Autre**	Other	Other	factor
**Si autre, merci de préciser**	If other, please specify		character

Table S1 – List of chronic conditions presented to participants at inclusion and follow-ups.

Characteristic	2021			2022			2023		
	**With SARS-CoV-2 infection,** N = 262[1]	**Without SARS-CoV-2 infection,** N = 5,865[1]	**p-value[2]**	**With SARS-CoV-2 infection,** N = 1,131[1]	**Without SARS-CoV-2 infection,** N = 4,847[1]	**p-value[3]**	**With SARS-CoV-2 infection,** N = 589[1]	**Without SARS-CoV-2 infection,** N = 4,359[1]	**p-value[3]**
Health event - New diagnosis	6 (2.3%)	76 (1.3%)	0.2	37 (3.3%)	75 (1.5%)	<0.001	28 (4.8%)	91 (2.1%)	<0.001
Health event - Worsening conditions	5 (1.9%)	48 (0.8%)	0.075	22 (1.9%)	54 (1.1%)	0.025	12 (2.0%)	49 (1.1%)	0.059

[1]n (%)

[2]Fisher's exact test

[3]Pearson's Chi-squared test

Table S2 – Distribution of new chronic condition diagnoses and worsening of pre-existing conditions by SARS-CoV-2 infection status and year of follow-up.

**Variable**	**Overall**, N = 8,086[1]	**Complete**, N = 3,467[1]	**Dropout**, N = 3,138[1]	**Intermittent Missing**, N = 1,481[1]	**p-value**[2]
**Age - Baseline**					<0.001
18-34	1,200 (15%)	294 (8.5%)	662 (21%)	244 (16%)	
35-49	2,862 (35%)	984 (28%)	1,294 (41%)	584 (39%)	
50-64	2,721 (34%)	1,417 (41%)	867 (28%)	437 (30%)	
65-79	1,193 (15%)	730 (21%)	264 (8.4%)	199 (13%)	
80+	110 (1.4%)	42 (1.2%)	51 (1.6%)	17 (1.1%)	
**Sex - Baseline**					0.11
Male	3,335 (41%)	1,433 (41%)	1,325 (42%)	577 (39%)	
Female	4,751 (59%)	2,034 (59%)	1,813 (58%)	904 (61%)	
**Insurance deductible - Baseline**					<0.001
300 CHF	2,737 (34%)	1,195 (34%)	1,036 (33%)	506 (34%)	
500 CHF	1,347 (17%)	604 (17%)	509 (16%)	234 (16%)	
1,000 CHF	327 (4.0%)	144 (4.2%)	123 (3.9%)	60 (4.1%)	
1,500 CHF	564 (7.0%)	295 (8.5%)	190 (6.1%)	79 (5.3%)	
2,000 CHF	175 (2.2%)	71 (2.0%)	73 (2.3%)	31 (2.1%)	
2,500 CHF	2,267 (28%)	968 (28%)	873 (28%)	426 (29%)	
Don't know/don't wish to answer	502 (6.2%)	128 (3.7%)	265 (8.5%)	109 (7.4%)	
No Swiss health insurance	163 (2.0%)	62 (1.8%)	67 (2.1%)	34 (2.3%)	
Missing[3]	4	0	2	2	
**Education level - Baseline**					<0.001
Primary	370 (4.6%)	109 (3.1%)	196 (6.3%)	65 (4.4%)	
Secondary	2,511 (31%)	1,107 (32%)	980 (31%)	424 (29%)	
Tertiary	5,188 (64%)	2,247 (65%)	1,952 (62%)	989 (67%)	
Missing[3]	17	4	10	3	
**Work situation - Baseline**					<0.001
Salaried	5,146 (64%)	2,083 (60%)	2,141 (68%)	922 (62%)	
Freelance/sole trader	574 (7.1%)	268 (7.7%)	211 (6.7%)	95 (6.4%)	
Retired	1,379 (17%)	801 (23%)	330 (11%)	248 (17%)	
Unemployed	235 (2.9%)	71 (2.0%)	107 (3.4%)	57 (3.9%)	
Other economically inactive	750 (9.3%)	243 (7.0%)	349 (11%)	158 (11%)	
Missing[3]	2	1	0	1	
**Household income - Baseline**					<0.001
Low	1,194 (15%)	420 (12%)	552 (18%)	222 (15%)	
Middle	4,229 (52%)	1,952 (56%)	1,509 (48%)	768 (52%)	

Variable	Overall, N = 8,086[1]	Complete, N = 3,467[1]	Dropout, N = 3,138[1]	Intermittent Missing, N = 1,481[1]	p-value[2]
High	1,154 (14%)	534 (15%)	413 (13%)	207 (14%)	
Don't know/don't wish to answer	1,500 (19%)	561 (16%)	658 (21%)	281 (19%)	
Missing[3]	9	0	6	3	
**Occupation - Baseline**					<0.001
Blue collar workers	776 (9.6%)	261 (7.5%)	399 (13%)	116 (7.8%)	
Independent workers	166 (2.1%)	51 (1.5%)	67 (2.1%)	48 (3.2%)	
Higher-grade white collar workers	2,056 (25%)	934 (27%)	740 (24%)	382 (26%)	
Lower-grade white collar workers	2,014 (25%)	917 (26%)	747 (24%)	350 (24%)	
Professional-Managers	2,560 (32%)	1,178 (34%)	923 (29%)	459 (31%)	
Other	508 (6.3%)	123 (3.6%)	261 (8.3%)	124 (8.4%)	
Missing[3]	6	3	1	2	
**Nationality - Baseline**					<0.001
Foreigner	1,532 (19%)	464 (13%)	752 (24%)	316 (21%)	
Swiss	6,553 (81%)	3,003 (87%)	2,385 (76%)	1,165 (79%)	
Missing[3]	1	0	1	0	
**Living status - Baseline**					<0.001
With partner and kids	3,636 (45%)	1,382 (40%)	1,545 (49%)	709 (48%)	
With partner, without kids	2,073 (26%)	1,106 (32%)	627 (20%)	340 (23%)	
Cohabitation	645 (8.0%)	178 (5.1%)	337 (11%)	130 (8.8%)	
Single parent	547 (6.8%)	222 (6.4%)	206 (6.6%)	119 (8.0%)	
Single	1,180 (15%)	579 (17%)	420 (13%)	181 (12%)	
Missing[3]	5	0	3	2	
**Chronic condition - Baseline**	2,041 (25%)	933 (27%)	723 (23%)	385 (26%)	0.001
Missing[3]	2	0	2	0	
**Forgoing healthcare - Baseline**	673 (8.3%)	305 (8.8%)	253 (8.1%)	115 (7.8%)	0.4
Missing[3]	3	0	1	2	

[1]Note: Participation patterns are defined as follows: Complete: Participated in all follow-ups; Dropout: Missing the final follow-up; Intermittent Missing: Present in last follow-up, but missed some in between

[2]Pearson's Chi-squared test

[3]Missing items were imputed using multiple imputation with chained equations (mice).

Table S3 – Comparison of baseline characteristics across participation patterns.

Variable	Odds ratio (95% CI)	Adjusted P-value	Variable group
**Base**			
1. SARS-CoV-2 infection (ref:no)	1.79 (1.10-2.92)	0.032*	Base
1. # of SARS-CoV-2 infections	1.24 (0.83-1.86)	0.294	Base
**Methodological**			
2. Preexisting chronic disease (baseline)	1.98 (1.54-2.55)	< 0.001***	1 + Methodological
2. SARS-CoV-2 infection (ref:no)	1.94 (1.19-3.17)	0.015*	1 + Methodological
2. # of SARS-CoV-2 infections	1.13 (0.75-1.70)	0.622	1 + Methodological
**Sociodemographic**			
3. Preexisting chronic disease (baseline)	2.18 (1.68-2.81)	< 0.001***	2 + Sociodemographic
3. SARS-CoV-2 infection (ref:no)	1.95 (1.19-3.18)	0.019*	2 + Sociodemographic
3. # of SARS-CoV-2 infections	1.07 (0.71-1.60)	0.888	2 + Sociodemographic
**Socioeconomic**			
4. Preexisting chronic disease (baseline)	2.08 (1.61-2.69)	< 0.001***	3 + Socioeconomic
4. SARS-CoV-2 infection (ref:no)	1.93 (1.18-3.14)	0.048*	3 + Socioeconomic
4. # of SARS-CoV-2 infections	1.09 (0.73-1.64)	0.883	3 + Socioeconomic
**Healthcare access**			
5. Forgoing care (baseline)	2.02 (1.44-2.84)	< 0.001***	4 + Healthcare access
5. Preexisting chronic disease (baseline)	1.92 (1.48-2.50)	< 0.001***	4 + Healthcare access
5. SARS-CoV-2 infection (ref:no)	1.92 (1.17-3.13)	0.055	4 + Healthcare access
5. # of SARS-CoV-2 infections	1.09 (0.72-1.63)	0.903	4 + Healthcare access

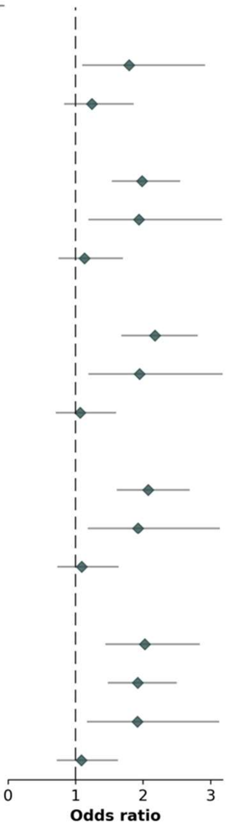

Figure S1 - Forest plot of odds ratios for factors associated with new chronic condition diagnosis. The plot shows adjusted odds ratios and 95% confidence intervals from a generalized linear mixed-effects model. The primary exposures of interest, SARS-CoV-2 infection (binary) and cumulative number of SARS-CoV-2 infections, are highlighted at the top. Other variables include sociodemographic factors and baseline health characteristics. Odds ratios greater than 1 indicate increased odds of the outcome, while those less than 1 indicate decreased odds. The dotted vertical line represents an odds ratio of 1 (no association).

Variable	Category	Odds ratio (95% CI)	Adjusted P-value	Variable group
**Base**				
1. SARS-CoV-2 infection (ref:no)		2.11 (1.24-3.59)	0.012*	Base
**Methodological**				
2. SARS-CoV-2 infection (ref:no)		2.08 (1.04-4.14)	0.172	1 + Methodological
2. SARS-CoV-2 infection:Missingness Pattern	Intermittent Missing	1.44 (0.38-5.45)	0.705	1 + Methodological
2. SARS-CoV-2 infection:Missingness Pattern	Dropout	0.77 (0.21-2.91)	0.705	1 + Methodological
**Sociodemographic**				
3. SARS-CoV-2 infection (ref:no)		2.12 (1.06-4.25)	0.249	2 + Sociodemographic
3. SARS-CoV-2 infection:Missingness Pattern	Intermittent Missing	1.48 (0.39-5.64)	0.748	2 + Sociodemographic
3. SARS-CoV-2 infection:Missingness Pattern	Dropout	0.80 (0.21-3.04)	0.748	2 + Sociodemographic
**Socioeconomic**				
4. SARS-CoV-2 infection (ref:no)		2.16 (1.07-4.36)	0.205	3 + Socioeconomic
4. SARS-CoV-2 infection:Missingness Pattern	Intermittent Missing	1.81 (0.46-7.07)	0.722	3 + Socioeconomic
4. SARS-CoV-2 infection:Missingness Pattern	Dropout	0.77 (0.20-2.94)	0.811	3 + Socioeconomic
**Healthcare access**				
5. Forgoing care (baseline)		1.98 (1.20-3.28)	0.086	4 + Healthcare access
5. SARS-CoV-2 infection (ref:no)		1.92 (1.03-3.56)	0.254	4 + Healthcare access
5. SARS-CoV-2 infection:Missingness Pattern	Intermittent Missing	1.45 (0.45-4.66)	0.767	4 + Healthcare access
5. SARS-CoV-2 infection:Missingness Pattern	Dropout	0.87 (0.27-2.78)	0.853	4 + Healthcare access

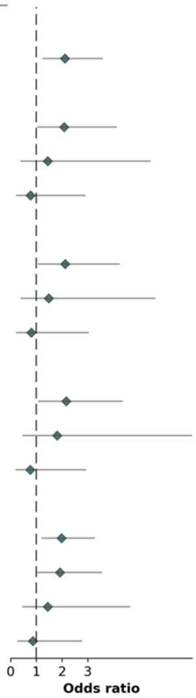

Figure S2. Forest plot of odds ratios for the association between SARS-CoV-2 infections and worsening chronic conditions. The plot shows adjusted odds ratios and 95% confidence intervals from generalized linear mixed-effects models (Model 1 to 5). Odds ratios greater than 1 indicate increased odds of the outcome, while those less than 1 indicate decreased odds. The dotted vertical line represents an odds ratio of 1 (no association).
